# Implication of the Whitefly Protein Vps Twenty Associated 1 (Vta1) in the Transmission of Cotton Leaf Curl Multan Virus

**DOI:** 10.3390/microorganisms9020304

**Published:** 2021-02-02

**Authors:** Yao Chi, Li-Long Pan, Shu-Sheng Liu, Shahid Mansoor, Xiao-Wei Wang

**Affiliations:** 1 Ministry of Agriculture Key Laboratory of Molecular Biology of Crop Pathogens and Insects, Institute of Insect Sciences, Zhejiang University, Hangzhou 310058, China; 21616153@zju.edu.cn (Y.C.); panlilong@zju.edu.cn (L.-L.P.); shshliu@zju.edu.cn (S.-S.L.); 2 National Institute for Biotechnology and Genetic Engineering, Faisalabad 38000, Pakistan; shahidmansoor7@gmail.com

**Keywords:** whitefly, begomovirus, Vta1, virus transmission, coat proteins

## Abstract

Cotton leaf curl Multan virus (CLCuMuV) is one of the major casual agents of cotton leaf curl disease. Previous studies show that two indigenous whitefly species of the *Bemisia tabaci* complex, Asia II 1 and Asia II 7, are able to transmit CLCuMuV, but the molecular mechanisms underlying the transmission are poorly known. In this study, we attempted to identify the whitefly proteins involved in CLCuMuV transmission. First, using a yeast two-hybrid system, we identified 54 candidate proteins of Asia II 1 that putatively can interact with the coat protein of CLCuMuV. Second, we examined interactions between the CLCuMuV coat protein and several whitefly proteins, including vacuolar protein sorting-associated protein (Vps) twenty associated 1 (Vta1). Third, using RNA interference, we found that Vta1 positively regulated CLCuMuV acquisition and transmission by the Asia II 1 whitefly. In addition, we showed that the interaction between the CLCuMuV coat protein and Vta1 from the whitefly Middle East-Asia Minor (MEAM1), a poor vector of CLCuMuV, was much weaker than that between Asia II 1 Vta1 and the CLCuMuV coat protein. Silencing of *Vta1* in MEAM1 did not affect the quantity of CLCuMuV acquired by the whitefly. Taken together, our results suggest that Vta1 may play an important role in the transmission of CLCuMuV by the whitefly.

## 1. Introduction

Plant viruses pose considerable threats to the production of many crops in modern agriculture [1]. The majority of plant viruses are transmitted by insect vectors (vector borne) [2]. In the past decades, geminiviruses, a subgroup of vector-borne plant viruses, have caused extensive epidemics in many crops, most notably in developing countries [3]. Among the nine genera in the family *Geminiviridae*, *Begomovirus* is the largest genus, containing over 400 species [4,5]. Begomoviruses are transmitted by whiteflies of the *Bemisia tabaci* complex, which comprises over 40 cryptic species, in a persistent circulative manner [2,3,6].

So far, studies concerning whitefly transmission of begomoviruses have been mostly conducted with tomato yellow leaf curl viruses (TYLCV). As learned from these studies, once TYLCV is acquired by the whitefly, the virus goes through the food canal to reach the filter chamber, from where it crosses the midgut wall and reaches the whitefly hemolymph; the virus then infects whitefly primary salivary glands and is secreted with saliva during feeding [7]. During this process, TYLCV hijacks clathrin-mediated endocytosis and the endosomal network to cross the midgut barrier of the whitefly vector [8,9]. Moreover, the coat protein (CP) of TYLCV may interact with many whitefly proteins, thereby facilitating virus transmission [3,10,11,12]. Two recent reviews on whitefly transmission of begomoviruses indicate that the transmission efficiency of a given virus may vary with different whitefly species, and different viruses may be transmitted with disparate efficiencies by a given whitefly species [3,10]. These variations indicate that the transmission mechanisms among different whitefly–begomovirus combinations may vary, highlighting the need for unravelling transmission mechanisms with previously unexplored whitefly species or begomoviruses.

Cotton leaf curl Multan virus (CLCuMuV) is one of the major casual agents of cotton leaf curl disease, one of the most significant constraints in cotton production in South Asia [13]. CLCuMuV was the major virus causing cotton leaf curl disease in South Asia in the 1990s and seemed to have been displaced by the Burewala strain of the cotton leaf curl Kokhran virus (CLCuKoV-Bur) at the beginning of this century [13,14]. In recent years, however, field surveys in India have revealed the rebound of CLCuMuV and the association of the recombinant variants of this virus with the breakdown of resistance in cotton [15,16]. The field surveys also indicate that in some regions in northwest India, CLCuMuV became the dominant virus in the cotton field, a sign of displacement of CLCuKoV-Bur by CLCuMuV [17].

Laboratory studies on the transmission of CLCuMuV by different whitefly species show that the virus can be efficiently transmitted by Asia II 1 and Asia II 7, two indigenous species of whiteflies from Asia, but can be hardly transmitted by other species of whiteflies, including MEAM1, Mediterranean (MED) and Asia 1 [18,19]. In this study, first we used split-ubiquitin yeast two-hybrid assay to identify Asia II 1 whitefly proteins that putatively interact with the CP of CLCuMuV. Next, we used yeast two-hybrid and pull-down assay to detect the interaction between CLCuMuV CP and several putative whitefly proteins, including vacuolar protein sorting-associated protein (Vps) twenty associated 1 (Vta1). We then used RNA interference to investigate the role of Vta1 in the acquisition and transmission of CLCuMuV by Asia II 1. In addition, we examined the function of Vta1 in MEAM1, a poor vector of CLCuMuV, in its transmission of the virus. Our findings provide new insights into the transmission of CLCuMuV by whiteflies.

## 2. Material and Methods

### 2.1. Plants, Insects, and Viruses

For plants, cotton (*Gossypium hirsutum* L. cv. Zhemian 1793 and Xinhai 21) and tobacco (*Nicotiana tabacum* L. cv. NC89) were used. Plants were grown in insect-proof greenhouses under natural lighting at controlled temperatures of 25 ± 3 °C and 14 h light/10 h darkness. Infectious clones of CLCuMuV isolate GD37 (GenBank accession number: JN968573) with its conjugated beta-satellite (GenBank accession number: JN968574) were introduced into 3–4 true-leaf-stage tobacco plants. Next, Asia II 1 transmission was used to obtained CLCuMuV-infected tobacco plants. Infection of plants was verified by symptom inspection and PCR detection of the virus using primers CLCuMuV-PCR-F and CLCuMuV-PCR-R (Table S1).

For insects, two whitefly species, namely Asia II 1 (mt*COI* GenBank accession number: DQ309077) and MEAM1 (mt*COI* GenBank accession number: KM821540), were used. Cultures of the two species were originally established from whiteflies collected from the field and have been maintained on cotton plants (cv. Zhemian 1793). Maintenance of whitefly cultures and all experiments were conducted in climate chambers at 26 ± 2 °C, in 14 h light/10 h darkness, and at 60–80% relative humidity. The purity of each of the whitefly cultures was monitored every 2 months using the mt*COI* PCR-RFLP technique and sequencing, as previously reported [20]. All female adult whiteflies were within 7 days post-emergence when used in experiments. 

### 2.2. Yeast Two-Hybrid System

The split-ubiquitin yeast two-hybrid system (Dualsystems Biotech, Zurich, Switzerland) was used to identify Asia II 1 whitefly proteins that interact with the CLCuMuV CP [11]. A cDNA library of Asia II 1 whitefly was constructed in the prey plasmid, SfiI-digested pPR3-N, with the EasyClone cDNA library construction kit (Dualsystems Biotech, Zurich, Switzerland). The quality of the cDNA library was determined as per the kit manual. The titer of the cDNA library of Asia II 1 was over 2 × 10^6^ cfu/19.5 μL, with an average insert size of over 1.0 kb, meeting the requirements of a standard cDNA library. The CLCuMuV CP gene was ligated into the bait plasmid pDHB1 using primers CLCuMuV-CP-pDHB1-infusion-F and CLCuMuV-CP-pDHB1-infusion-R (Table S1). The recombinant plasmid pDHB1-CLCuMuV CP was introduced into yeast strain NMY51, and the expression of the CLCuMuV CP in yeast was verified by Western blotting using anti-TYLCV CP mouse monoclonal antibodies (mAb) (provided by Professor Xue-Ping Zhou, Institute of Biotechnology, Zhejiang University). Next, the cDNA library was introduced into yeast cells containing the pDHB1-CLCuMuV CP. Yeast clones were selected on triple dropout (TDO) medium (S.D./-His/-Leu/-Trp) containing 2.5 mM of 3-aminotriazole (3-AT). The yeast cells were then resuspended in 0.9% NaCl solution (to OD600 = 1.0) and later restreaked on quadruple dropout (QDO) medium (S.D./-Ade/-His/-Leu/-Trp) containing 2.5 mM of 3-AT to verify interactions. In addition, a yeast beta-Gal assay kit (Thermo Scientific, Waltham, MA, USA) was used to examine the interactions by detecting beta-galactosidase activity in yeast clones. Finally, plasmids were recovered from yeast and transformed into *Escherichia. coli* strain DH5α and then sequenced. 

For the verification of interaction, plasmids recovered from yeast clones were transformed into yeast cells containing the pDHB1-CLCuMuV CP using the method described above. The full length of whitefly genes was cloned into the plasmid pPR3-N with primers Vta1-pPR3-N-infusion-F, Vta1-pPR3-N-infusion-R, pPR3-N-infusion-F, and pPR3-N-infusion-R (Table S1) and analyzed using procedures as described above.

### 2.3. Bioinformatics Analysis

The whitefly genes whose coding proteins were verified to interact with the CLCuMuV CP were annotated using BLAST (http://blast.st-va.ncbi.nlm.nih.gov/Blast.cgi). Gene Ontology (GO) enrichment analysis was then performed using the OmicShare tools (http://www.omicshare.com/tools). 

### 2.4. Cloning of Vta1 in Asia II 1 and MEAM1

The predicted full length of MEAM1 *Vta1* was found on the NCBI database (Genbank accession code: LOC109029924). Therefore, we cloned MEAM1 *Vta1* and submitted it to the NCBI database under accession number NW380743. The full length of *Vta1* in the Asia II 1 whitefly was amplified by the SMARTer RACE 5′/3′ kit (Clontech, Kyoto Japan), as per the manufacturer’s protocol, with primers 5′ race-Vta1, 5′ race-Vta1, and 3′ race-Vta1-CS1 (Table S1). Total RNAs extracted from the Asia II 1 whitefly using TRIzol reagent (Ambion, Waltham, MA, USA) were used in RACE. Next, Asia II 1 *Vta1* was cloned and submitted to the NCBI database under accession number MW346674.

### 2.5. Pull-Down Assay

The full length of the CLCuMuV CP gene was cloned into pGEX-6p-1 for fusion with glutathione S-transferase (GST) using primers CLCuMuV-CP-pGEX-6p-1-F and CLCuMuV-CP-pGEX-6p-1-R (Table S1). The full length of *Vta1* was cloned into pMAL-c5x for fusion with maltose-binding protein (MBP) using primers Vta1-pMAL-c5x-F and Vta1-pMAL-c5x-R (Table S1). Recombinant proteins were expressed in *E. coli* strain BL21. After purification, the GST-CLCuMuV CP and GST (control) were allowed to bind to glutathione agarose beads (GE Healthcare, Boston, MA, USA) for 2 h at 4 °C. The beads were then washed and incubated with MBP-Vta1 or MBP (control) at 4 °C for 4 h. Next, the beads were washed and boiled, and the bead-bound proteins were separated using SDS-PAGE and detected using Western blotting with anti-MBP rabbit polyclonal antibodies (pAb) (Abcam, Cambridge, UK). 

### 2.6. Double Strand RNA (DsRNA) Synthesis and Membrane Feeding

DNA templates for dsRNA synthesis were amplified by PCR using primers with the T7 promoter at both ends, namely *Vta1* (Asia II 1)-T7-F, *Vta1* (Asia II 1)-T7-R, *Vta1* (MEAM1)-T7-F, and *Vta1* (MEAM1)-T7-R (Table S1). DsRNA synthesis was conducted with a T7 high-yield RNA transcription kit (Vazyme, Nanjing, China). Next, dsRNA was purified, and the quality and concentration were determined using agarose gel electrophoresis and Nanodrop (Thermo Fisher, Waltham, MA, USA). For membrane feeding, dsRNA-targeting *Vta1* or *GFP* (control) was added to 15% sucrose solution to make the final concentration 200 ng/μL. Whiteflies were collected and released into artificial diet feeding chambers, as described before [8]. The duration of membrane feeding was 48 h.

### 2.7. Analysis of Gene Expression Level

Total RNAs of the whitefly were extracted using TRIzol reagent as per the manufacturer’s instructions. cDNA was synthesized from 1 μg of RNA using the PrimeScript RT reagent kit (Takara, Kyoto, Japan). qPCR was performed on the CFX96™ Real-Time PCR Detection System (Bio-Rad, Hercules, CA, USA) with SYBR Premix Ex Taq II (TaKaRa, Kyoto, Japan). Primers β-actin-qPCR-F and β-actin-qPCR-R were used as internal controls. Vta1 (Asia II 1)-qPCR-F and Vta1 (Asia II 1)-qPCR-R were used for Asia II 1 *Vta1*, and Vta1 (MEAM1)-qPCR-F and Vta1 (MEAM1)-qPCR-R were used for MEAM1 *Vta1*. All primes are listed in Table S1.

### 2.8. Virus Acquisition and Quantification of CLCuMuV in the Whitefly

For virus acquisition, whiteflies were collected and allowed to feed on CLCuMuV-infected tobacco plants. Forty-eight hours later, virus quantification was performed. For the whitefly whole body, whitefly adults were collected as groups of 15 and lysed in lysis buffer (50 mM KCl, 10 mM Tris, 0.45% Tween 20, 0.2% gelatin, 0.45% NP40, 60 mg/mL proteinase K, with pH 8.4). As for organs, 4 midguts or primary salivary glands were collected as one sample, and hemolymph from 4 whiteflies was treated as one sample. The collection and preparation of whitefly organs were conducted, as described before [18]. qPCR was conducted, as described above, with primers β-actin-qPCR-F, β-actin-qPCR-R, CLCuMuV-qPCR-F, and CLCuMuV-qPCR-R (Table S1). 

### 2.9. Virus Transmission

Plants of tobacco (cv. NC89) and cotton (cv. Xinhai 21) were used. When tobacco plants were used, female adult whiteflies that had fed on virus-infected plants for 48 h were collected as groups of 10 and then transferred to be placed on a 3–4 true-leaf-stage tobacco seedling using clip cages [21] to feed for 48 h. Three replicates were conducted, with each containing 6–10 plants. The whiteflies were then removed, and the plants were sprayed with imidacloprid (20 mg/L) to kill all the eggs. The infection status of test plants was examined by symptom inspection and PCR detection of viral DNAs, as described above, 30 days post-inoculation.

When cotton plants were used, female adult whiteflies that had fed on virus-infected plants for 72 h were collected as groups of 10 and then transferred to feed on a 1–2 true-leaf-stage cotton seedling (enclosed in leaf-clip cages) for 72 h. Three replicates were conducted, with each containing 6–10 plants of both tobacco and cotton. After that, observations were conducted using the same procedure as that for tobacco except that the infection status of the plants was conducted 70 days post-inoculation.

### 2.10. Statistical Analysis

All qPCR data were calculated using 2^−△Ct^ as normalized to whitefly *actin*. For the comparison of gene expression level and quantity of viruses, an independent *t*-test was used. For the comparison of transmission efficiency, percentage data were arcsine-square-root-transformed for statistical analysis using an independent *t*-test and back-transformed for presentation. All statistical analyses were conducted using SPSS 20.0 Statistics (IBM, Armonk, NY, USA) and Microsoft Excel (version 2016, Microsoft, Redmond, WA, USA).

## 3. Results

### 3.1. Identification of Asia II 1 Whitefly Proteins That Interact with the CLCuMuV CP

We used the split-ubiquitin yeast two-hybrid system to identify proteins in the Asia II 1 whitefly that potentially interact with the CLCuMuV CP. The expression of the bait plasmid pDHB1-CLCuMuV CP in yeast was verified using Western blotting (Figure S1), showing the functionality of the bait plasmid pDHB1-CLCuMuV CP. After screening of the Asia II 1 whitefly cDNA library using this yeast two-hybrid system, more than 300 positive clones were isolated and the sequencing results indicated that plasmids in these positive clones encode 200 unique proteins. To examine the interactions between the CLCuMuV CP and whitefly proteins encoded by the plasmids recovered from positive clones, we chose 70 proteins to examine their interaction with the CLCuMuV CP using the yeast two-hybrid system. Of these proteins, 54 were found to interact with the CLCuMuV CP. A BLAST search of the NCBI database was then conducted for the 54 proteins, and the names of the protein sequences that share the highest homology were obtained (Table 1).

### 3.2. Bioinformatics Analysis of Whitefly Proteins That Interact with the CLCuMuV CP

**Figure 1:**
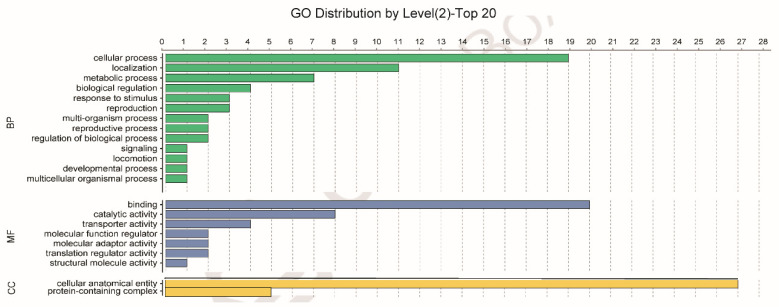
Gene Ontology (GO) analysis of the 54 Asia II 1 whitefly proteins that putatively interact with the CLCuMuV CP, as indicated by the yeast two-hybrid system. Different colors represent different GO categories (BP: biological process; MF: molecular function; CC: cellular components). GO annotation was conducted using Blast2GO software, and the figure was generated using OmicShare tools (http://www.omicshare.com/tools).

Using GO analysis, the whitefly proteins that putatively interact with the CLCuMuV CP were assigned to 13 biological processes (BPs) or seven molecular functions (MFs) or two cellular components (CCs) (Figure 1). Specifically, of the 13 BPs, cellular process contained the majority of proteins (19). Further analysis of these proteins indicated that some proteins can be further classified into vesicle-mediated transport (GO: 0016192), including calmodulin (CALM), vesicle transport protein SEC22 (SEC22), vacuolar protein sorting-associated protein (Vps) twenty associated 1 (Vta1), Ras-related protein Rab-6A (RAB6A), and vesicle-associated membrane protein 7 (VAMP7).

### 3.3. Verification of Interaction Using Yeast Two-Hybrid and Pull-Down Systems

**Figure 2:**
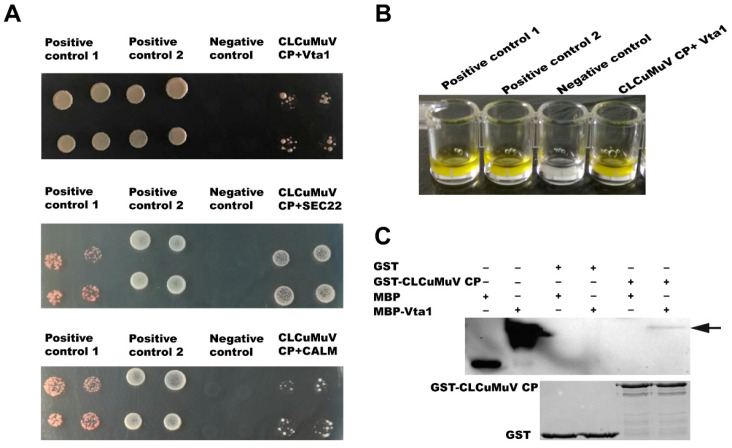
Verification of interaction between the CLCuMuV CP and whitefly proteins. The interaction between the full length of calmodulin (CALM), vesicle transport protein SEC22 (SEC22), and vacuolar protein sorting-associated protein (Vps) twenty associated 1 (Vta1) with the CLCuMuV CP in yeast (**A**). Positive control 1: pDHB1-CLCuMV-CP+pOst1-NubI; positive control 2: pDHB1-large T+pDSL- p53; negative control: pDHB1-CLCuMV-CP+pPR3-N. Beta-gal activity in yeast cells (**B**). Pull-down assay between CLCuMuV CP and Vta1 (**C**).

For verification of the interaction between the CLCuMuV CP and prey proteins, we selected the five proteins in the GO category vesicle-mediated transport (GO:0016192) for further analysis using the yeast two-hybrid system. The full-length open reading frames of the five genes were amplified and ligated into pPR3-N, and then these prey plasmids and the bait plasmid pDHB1-CLCuMuV CP were co-transformed into yeast cells. Among the five proteins, the full lengths of calmodulin (CALM), vesicle transport protein SEC22 (SEC22), and vacuolar protein sorting-associated protein (Vps) twenty associated 1 (Vta1) were found to interact with the CLCuMuV CP (Figure 2A). Since Vta1 has been reported to regulate virus–host interactions, we subjected it to further analysis [22,23]. Analysis of beta-gal activity confirmed the interaction between Vta1 and the CLCuMuV CP (Figure 2B). And in the pull-down assay, when the fusion protein GST-CLCuMuV CP was used as a bait protein and the fusion protein MBP-Vta1 used as the prey protein, the prey protein could co-elute with the GST-fused CLCuMuV CP (Figure 2C). These results suggest that Vta1 from Asia II 1 whitefly can interact with the CLCuMuV CP both in vivo and in vitro.

### 3.4. Functional Characterization of Vta1 in Asia II 1 Transmission of CLCuMuV

**Figure 3:**
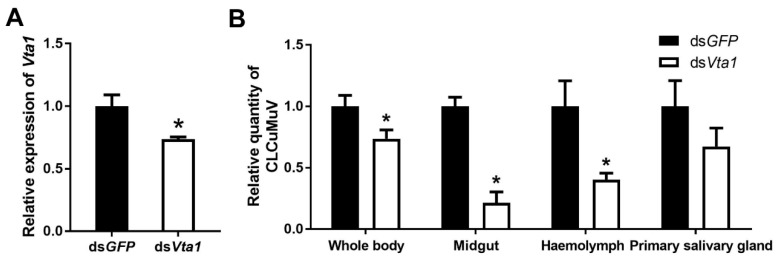
Effects of *Vta1* knockdown on CLCuMuV acquisition by Asia II 1. Gene expression level of *Vta1* following dsRNA feeding (*n* = 4 for ds*Vta1* or ds*GFP*) (**A**). Following feeding, virus quantity in whitefly whole body and organs (*n* = 3–8 for whole body and midgut, 19–22 for hemolymph, and 12 for primary salivary glands) (**B**). * stands for significant difference (independent *t*-test, *p* < 0.05).

**Figure 4:**
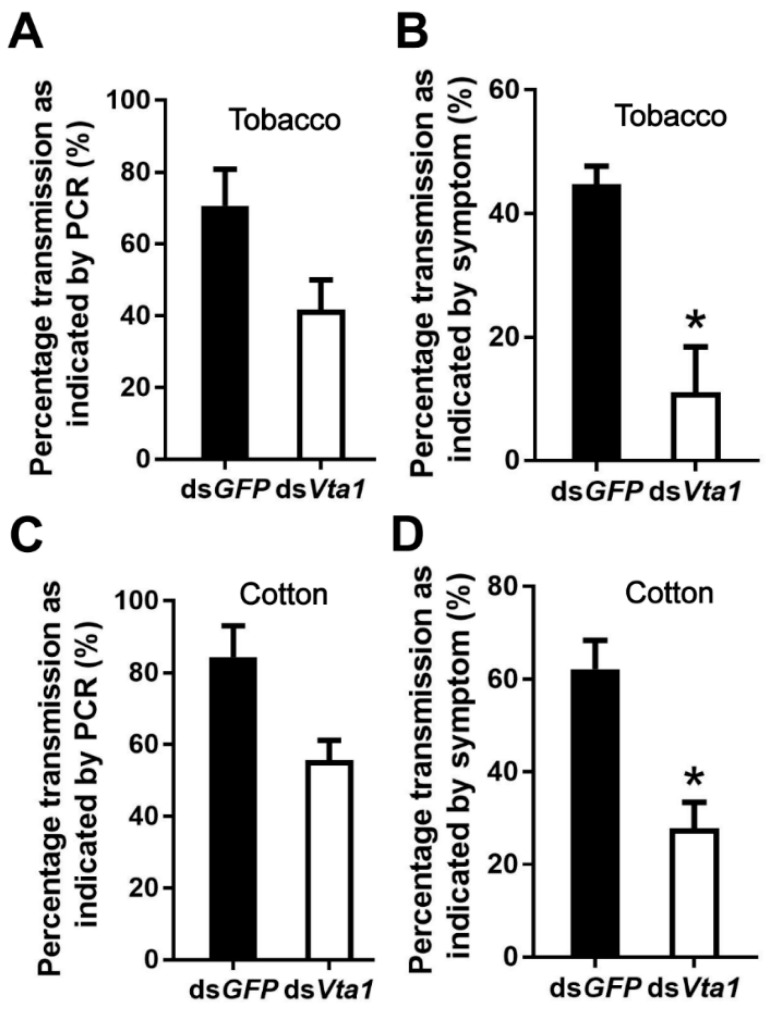
Effects of *Vta1* knockdown on CLCuMuV transmission by Asia II 1. Whiteflies that had acquired CLCuMuV were collected and transferred to feed on tobacco (**A**,**B**) or cotton (**C**,**D**). On each of the two test plants, 3 replicates were conducted for both ds*GFP* and ds*Vta1*, with each replicate containing 6–10 plants. * stands for significant difference (independent *t*-test, *p* < 0.05).

To examine the function of Vta1, Asia II 1 whiteflies were fed with *Vta1* dsRNA. Following dsRNA feeding, the expression of *Vta1* in whiteflies was down-regulated by 27.4% as compared to controls (Figure 3A). Next, the whiteflies were transferred to feed on CLCuMuV-infected tobacco plants for 48 h for virus acquisition. Knockdown of *Vta1* resulted in a significant decrease in the relative virus quantity in the whiteflies’ whole body, midgut, and hemolymph but did not cause a significant change in the relative virus quantity in primary salivary glands (Figure 3B). Further, transmission trails were performed. When tobacco plants were used as test plants, knockdown of *Vta1* resulted in a significant decrease in CLCuMuV transmission, as shown by percentages of plants with viral symptoms but not by viral detection using PCR (Figure 4A,B). Similar results were found when cotton plants were used as test plants (Figure 4C,D). These results suggest that *Vta1* plays an important role in CLCuMuV acquisition and transmission by Asia II 1.

### 3.5. The Role of Vta1 in CLCuMuV Transmission by MEAM1

**Figure 5:**
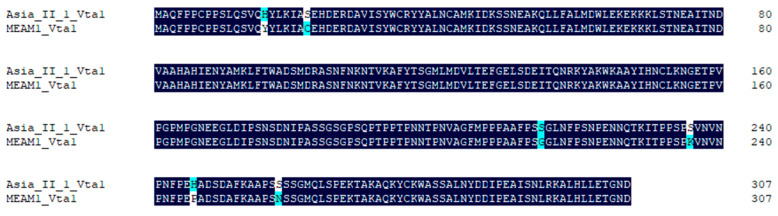
Comparison of amino acid sequences of Asia II 1 Vta1 and MEAM1 Vta1. Alignments were performed using DNAMAN. Dark blue indicates consensus in amino acids between the two sequences, and light blue or white indicates divergence in amino acids.

**Figure 6:**
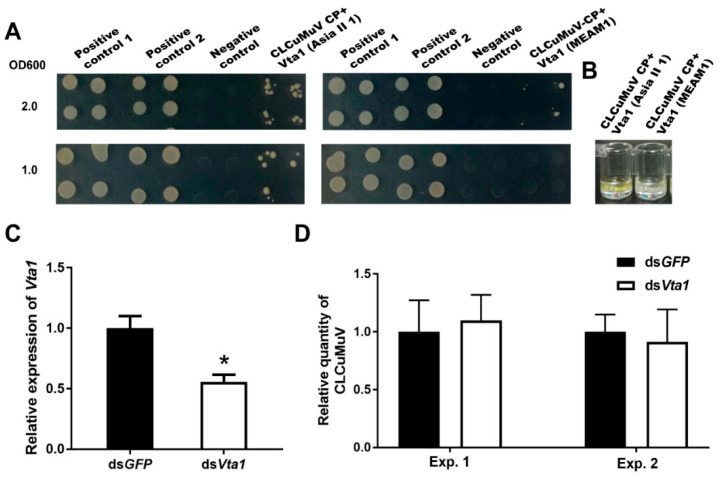
The role of *Vta1* in CLCuMuV transmission by MEAM1. The yeast two-hybrid assay was performed to compare the affinity of the CLCuMuV CP to Asia II 1 Vta1 and MEAM1 Vta1. Yeast cells containing different combination of plasmids were resuspended to OD600 being 1.0 and 2.0 and then cultured on quadruple dropout medium containing 2.5 mM of 3-AT (**A**). Positive control 1: pDHB1-CLCuMV-CP+pOst1-NubI; positive control 2: pDHB1-large T+pDSL-p53; negative control: pDHB1-CLCuMV-CP+pPR3-N. Yeast beta-gal assay kit was used to examine the interactions (**B**). Further, knockdown of MEAM1 *Vta1* was performed, and the whiteflies were then set to acquire CLCuMuV for 48 h. MEAM1 *Vta1* expression level (*n* = 4 for ds*GFP* or ds*Vta1*) (**C**), and quantity of CLCuMuV in whitefly whole body in two experiments (*n* = 4–5 for ds*GFP* or ds*Vta1* in each experiment) (**D**). * stands for significant difference (independent *t*-test, *p* < 0.05 for **C**,**D**).

Asia II 1 was able to readily transmit CLCuMuV, while MEAM1 can only transmit this virus with very low efficiency [18]. To explore whether *Vta1* plays a role in CLCuMuV transmission by MEAM1, we first compared the amino acid sequence of MEAM1 Vta1 with that of Asia II 1 Vta1 and found that six amino acids are different between the two Vta1s (Figure 5). The yeast two-hybrid system was then used to compare Asia II 1 Vta1 and MEAM1 Vta1 in interaction with the CLCuMuV CP. Yeast cells were resuspended to certain ODs and then cultured on quadruple dropout medium (SD/-Leu/-Trp/-His/-Ade) containing 2.5 mM of 3-AT. When OD600 was 1.0, yeast cells containing the pDHB1-CLCuMuV CP and pPR3-N-MEAM1 Vta1 did not grow, but cells containing pDHB1-CLCuMuV CP and pPR3-N-Asia II 1 Vta1 grew to noticeable colonies. To further determine whether there is any detectable interaction between the CLCuMuV CP and MEAM1 Vta1, we adjusted OD600 to 2.0. Under this condition, yeast cells containing the pDHB1-CLCuMuV CP and pPR3-N-MEAM1 Vta1 grew to colonies, but they were much smaller than those containing the pDHB1-CLCuMuV CP and pPR3-N-Asia II 1 Vta1 (Figure 6A). Analysis of beta-gal activity in yeast cells containing different combination of plasmids revealed that the interaction between MEAM1 Vta1 and the CLCuMuV CP was very weak, if any, as judged by the unappreciable yellow color in the solution (Figure 6B). Knockdown of MEAM1 *Vta1* resulted in significant down-regulation of the *Vta1* expression level by 44.4% (Figure 6C). Following virus acquisition, knockdown of *Vta1* did not change the quantity of CLCuMuV acquired by MEAM1 in two independent experiments (Figure 6D). These results suggest that *Vta1* plays a minor, if any, role in CLCuMuV transmission by the MEAM1 whitefly.

## 4. Discussion

In this study, using the yeast two-hybrid system, we identified 54 candidate Asia II 1 proteins that putatively interact with the CLCuMuV CP (Table 1). GO enrichment analysis showed that these proteins may be responsible for 13 different biological processes (Figure 1). Based on the molecular function of the identified proteins, we selected five proteins to verify their interactions with the CLCuMuV CP using the yeast two-hybrid system and confirmed the interaction of Vta1 with the CLCuMuV CP using the GST pull-down assay (Figure 2). Next, we found that RNA interference of *Vta1* in Asia II 1 reduced the virus quantity in the whitefly and the efficiency of virus transmission (Figure 3 and Figure 4). It should be noted that decreases in virus quantity were significant in the whitefly midgut and hemolymph but not in primary salivary glands upon *Vta1* silencing (Figure 3). This might be due to the fact that the silencing efficiency of *Vta1* is lower in primary salivary glands than that in the midgut and hemolymph. Moreover, knockdown of *Vta1* in Asia II 1 results in significant decreases in the virus transmission efficiency, as indicated by symptom inspection, but the decrease is not significant when examined by PCR (Figure 4). The possible reason for the discrepancy is that PCR is very sensitive, so it may amplify even trace amounts of viral DNAs in whitefly-inoculated plants. However, symptom appearance requires the accumulation of a substantial amount of viruses, which is a better indicator of successful inoculation. In addition, we showed that MEAM1 Vta1, the sequence of which is slightly different from that of Asia II 1 Vta1, exhibited much lower affinity to the CLCuMuV CP than Asia II 1 Vta1, and RNA interference of MEAM1 *Vta1* did not affect the quantity of virus acquired by MEAM1 (Figure 5 and Figure 6).

In eukaryotic cells, Vta1 functions as a cofactor of vacuolar protein sorting 4 (Vps4) by impacting its oligomerization, thereby regulating the activity of Vps4 to modulate endosomal sorting complexes required for transport (ESCRT) [24]. ESCRTs include ESCRT-0, ESCRT-I, ESCRT-II, and ESCRT-III and are involved in regulating the function of multivesicular bodies, which are an endosomal-membrane-trafficking and protein-sorting station [25]. In the context of virus–host or virus–vector interactions, the role of Vta1 is little known. The mammalian homologue of Vta1, LIP5, positively regulates the budding of human immunodeficiency virus type 1 in human cells [23]. In addition, deletion of MIT domains of *Spodoptera frugiperda* Vta1 reduces the replication of *Autographa californica* multiple nucleopolyhedrovirus [22]. In this study, we found that Vta1 positively regulates CLCuMuV transport across the midgut of Asia II 1 whiteflies following viral acquisition, as well as the efficiency of virus transmission. This may be due to the fact that Vta1 affects the ESCRT machinery via its action on Vps4, and the ESCRT machinery regulates vesicle trafficking, which has been shown to regulate whitefly transmission of begomoviruses [8,9]. Further, our recent findings show that the early endosome plays an important role in begomoviruses intracellular transport and an endocytic receptor can regulate vesicle transport of begomoviruses in whitefly midgut cells [9,12]. These results suggest that endosomal trafficking is important for the transport of both CLCuMuV and TYLCV across epithelial cells in the whitefly midgut. However, the unambiguous dissection of the role of endosomes and Vta1 in Asia II 1 transmission of CLCuMuV warrants further investigations.

For a given begomovirus, different whitefly species may transmit with disparate efficiencies [3,10]. Case studies have shown that this can be attributed to the differential capacity of the virus to cross barriers within the body of different whitefly species [18,26,27]. For example, the relatively high and low efficiencies in transmitting CLCuMuV by Asia II 1 and MEAM1 were found to be associated with the relatively high and low efficiencies of the virus to cross the midgut wall of the two species of whiteflies [18]. Here, we found that Vta1 from Asia II 1 positively regulates CLCuMuV transport across the midgut and transmission, and MEAM1 Vta1 does not seem to play a role in CLCuMuV transmission. Additionally, Asia II 1 Vta1 displays a stronger affinity to the CLCuMuV CP than MEAM1 Vta1. Hence, we propose that Vta1 may be a significant factor in determining the disparate efficiencies of CLCuMuV transmission by Asia II 1 and MEAM1, possibly through its different functions in facilitating the virus to cross physiological barriers such as the midgut in the vector body. Interestingly, a comparison of the amino acid sequences of MEAM1 Vta1 and Asia II 1 Vta1 showed that they differ in only six amino acids. The divergence at the six amino acids may directly contribute to the differential transmission of CLCuMuV by MEAM1 and Asia II 1. Of course, more empirical studies are needed to verify this hypothesis.

In summary, we identified 54 proteins from the Asia II 1 whitefly that putatively interact with the CLCuMuV CP. We showed that Asia II 1 Vta1 positively regulates the acquisition and transmission of CLCuMuV by the Asia II 1 whitefly. We further found that Vta1 from MEAM1, a poor vector of CLCuMuV, interacts with the CLCuMuV CP weakly and does not seem to play a role in CLCuMuV transmission by this whitefly. Taken together, our findings indicate that the protein Vta1 may be an important factor in determining the efficiency of CLCuMuV transmission by a given species of whitefly, and provide new insight into the interaction between single strand DNA (ssDNA) viruses and their insect vectors.

## Figures and Tables

**Table 1:** List of Asia II 1 proteins that putatively interact with the CLCuMuV CP as identified by the yeast two-hybrid system.

No.	Accession	Protein Name
1	XP_018912286.1	gelsolin-related protein of 125 kDa-like
2	XP_018897713.1	complement component 1 Q subcomponent-binding protein, mitochondrial isoform X2
3	XP_018906592.1	aquaporin AQPcic-like
4	XP_018902985.1	dnaJ homolog subfamily C member 7
5	XP_018896724.1	myosin regulatory light chain 2
6	XP_018913048.1	transmembrane protein 189
7	XP_018912886.1	biogenesis of lysosome-related organelles complex 1 subunit 5 isoform X4
8	XP_018902682.1	tubulin beta-1 chain
9	XP_018908832.1	vesicle-associated membrane protein 7 isoform X1
10	XP_018909667.1	ugar transporter SWEET1-like
11	XP_018905417.1	ornithine decarboxylase antizyme 1
12	XP_018903031.1	calmodulin isoform X1
13	XP_018896738.1	protein lifeguard 4-like
14	XP_018906525.1	hsp70-Hsp90 organizing protein 3-like
15	XP_018917594.1	glycosylphosphatidylinositol anchor attachment 1 protein
16	XP_018899531.1	vesicle-trafficking protein SEC22b
17	XP_018898178.1	chloride intracellular channel exc-4
18	XP_018901458.1	thioredoxin-2-like
19	XP_018911715.1	matrix metalloproteinase-14 isoform X1
20	XP_018905472.1	microtubule-associated protein futsch-like
21	XP_018899425.1	CD9 antigen
22	XP_018902269.1	protein YIPF6
23	XP_018917580.1	transport and Golgi organization protein 11 isoform X1
24	XP_018911339.1	translocation protein SEC62
25	XP_018900653.1	heat shock 70 kDa protein cognate 3
26	ADG03467.1	heat shock protein 20
27	XP_018903943.1	FAS-associated factor 1
28	XP_018914247.1	calcium-transporting ATPase sarcoplasmic/endoplasmic reticulum type isoform X1
29	XP_018910594.1	ABC transporter G family member 20-like
30	XP_018911729.1	dnaJ homolog subfamily C member 25-like
31	XP_018908958.1	heat shock 70 kDa protein cognate 4
32	XP_018903618.1	phosphate carrier protein, mitochondrial-like
33	XP_018907161.1	transmembrane protein 104 homolog
34	XP_018907534.1	elongation factor Tu-like
35	XP_018902665.1	vesicle transport through interaction with t-SNAREs homolog 1A
36	XP_018904067.1	vesicle-associated membrane protein 2-like isoform X1
37	XP_018908256.1	probable RNA polymerase II nuclear localization protein SLC7A6OS
38	XP_018896173.1	vacuolar protein sorting-associated protein vacuolar protein sorting-associated protein (Vps) twenty associated 1 homolog
39	XP_018898014.1	calreticulin
40	XP_018918053.1	microtubule-associated protein RP/EB family member 1-like
41	XP_018896547.1	protein jagunal
42	XP_018901184.1	nucleolar GTP-binding protein 2
43	XP_018901406.1	splicing factor 45
44	XP_018913448.1	ras-related protein Rab6
45	XP_018897712.1	complement component 1 Q subcomponent-binding protein, mitochondrial isoform X1
46	XP_018908611.1	uncharacterized protein LOC109038112
47	XP_018911543.1	uncharacterized protein LOC109040175
48	XP_018910467.1	uncharacterized protein LOC109039442 isoform X2
49	XP_018916418.1	uncharacterized protein LOC109043611
50	XP_018906808.1	uncharacterized protein LOC109036858
51	XP_018897050.1	uncharacterized protein LOC109030509
52	XP_018910780.1	uncharacterized protein LOC109039648
53	XP_018905235.1	uncharacterized protein LOC109035881
54	XP_018908284.1	uncharacterized protein LOC109037882

## Data Availability

Data in this study are available from the authors upon request.

## Supplementary Materials

The following are available online at https://www.mdpi.com/2076-2607/9/2/304/s1, Figure S1: The expression of pDHB1-CLCuMuV CP in yeast. Expression of CLCuMuV CP bait fusion protein was detected by western blot using anti-TYLCV CP antibodies; Table S1: Primers used in this study.
