# Supplementary material for: Implication of the Whitefly Protein Vps Twenty Associated 1 (Vta1) in the Transmission of Cotton Leaf Curl Multan Virus
Source: Microorganisms. 2021 Feb 2;9(2):304. doi: 10.3390/microorganisms9020304 (PMC7912986; doi:10.3390/microorganisms9020304)
Supplement: Supplementary file 1 [file microorganisms-09-00304-s001.pdf]

Table S1 Primers used in this study.

Primer Name	Sequence (5'-3')
CLCuMuV-PCR-F	TTCGGTGTATGCTTTCTGTC
CLCuMuV-PCR-R	ACCTGATAACGGTCCCTATG
CLCuMuV-CP-pDHB1-infusion-F	ATTACGGCCAGGCCTATGTCGAAGCGAGCTGCAGA
CLCuMuV-CP-pDHB1-infusion-R	CAAGATATACCATGGATTCGTTACAGAGTCATAAAAATA
pDHB1-infusion-F	CCATGGTATATCTTGGCCGC
pDHB1-infusion-R	AGGCCTGGCCGTAATGGC
Vta1-pPR3-N-infusion-F	TATCAACGCAGAGTGATGGCAACTTTACTGGATAAT
Vta1-pPR3-N-infusion-R	CCGGGCCGTAATGGCCTATGAATCATTTCCAGTCTC
pPR3-N-infusion-F	GCCATTACGGCCGCCGAAAA
pPR3-N-infusion-R	CACTCTGCGTTGATACCACT
5' race-Vta1	GATTACGCCAAGCTTGCATTCCAGAGGAACTACTGGGTGCAGC
3' race-Vta1	GATTACGCCAAGCTTGCCTCCTCCAGCTGCCTTTCCCTCTAGT
3' race-Vta1-CS1	GATTACGCCAAGCTTG CTGCACCCAG TAGTTCCTCT GGAATGC
CLCuMuV CP-pGEX-6p-1-F	CGCGGATCCATGTCGAAGCGAGCTGCAGAT
CLCuMuV CP-pGEX-6p-1-R	TCCCCCGGGTCAATTCGTTACAGAGTCATA
Vta1-pMAL-c5x-F	GGAATTCCATATGATGGCTCAATTTCCTCCCTGC
Vta1-pMAL-c5x-R	CGCGGATCCCTATGAATCATTTCCAGTCTC
Vta1 (Asia II 1)-T7-F	TAATACGACTCACTATAGGGAGACATGATGAAAGGGATGCTG
Vta1 (Asia II 1)-T7-R	TAATACGACTCACTATAGGGAGAGACCCGGAACTGGAGTCTCC
Vta1 (MEAM 1)-T7-F	TAATACGACTCACTATAGGGAGAGTCCAATGCCTGGTAATGAA
Vta1 (MEAM 1)-T7-R	TAATACGACTCACTATAGGGAGACTATGAATCATTTCCAGTCT
β-actin-qPCR-F	TCTTCCAGCCATCCTTCTTG
β-actin-qPCR-R	CGGTGATTTCCTTCTGCATT
Vta1 (Asia II 1)-qPCR-F	GGGTCTGGGCCAAGTCAACC
Vta1 (Asia II 1)-qPCR-R	AGGCAGCTGGAGGAGGCATA
Vta1 (MEAM 1)-qPCR-F	TGTTGCTGCACACGCCCATA
Vta1 (MEAM 1)-qPCR-R	ACGGTCCATACTGTCAGCCCATG
CLCuMuV-qPCR-F	ACACTTGTGCAGTCCCAGAG
CLCuMuV-qPCR-R	CACTTCAACCGTCCATTCAC

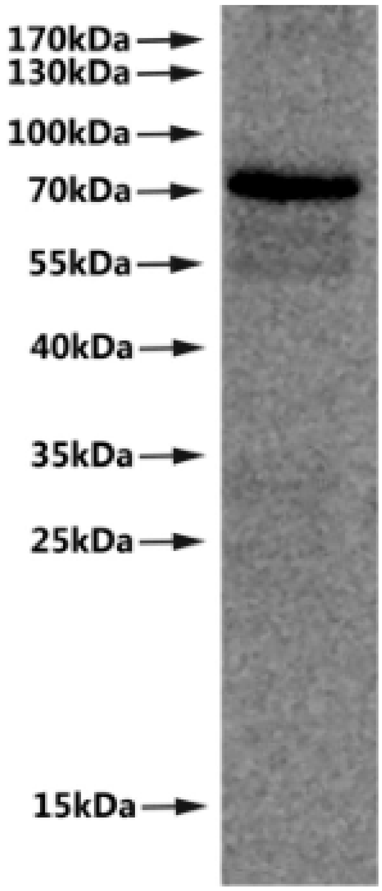

Figure. S1 The expression of pDHB1-CLCuMuV CP in yeast. Expression of CLCuMuV CP bait fusion protein was detected by western blot using anti-TYLCV CP antibodies.
